# Anterior Cruciate Ligament Reconstruction: Clinical Outcomes and Biomechanical Considerations of Graft Selection—A Narrative Review

**DOI:** 10.3390/medicina62091683

**Published:** 2026-09-02

**Authors:** Bogdan Huzum, Dan Viorel Cionca, Daniel Ciupilan, Cǎtǎlina Ionescu, Mihnea-Theodor Sîrbu, Malina Visternicu, Alin Ciobica, Oana Badulescu, Paul-Dan Sîrbu

**Affiliations:** 1 “Grigore T. Popa” University of Medicine and Pharmacy, University Street, No. 16, 700115 Iasi, Romania; bogdan.huzum@umfiasi.ro (B.H.); mihnea-theodor.sirbu@umfiasi.ro (M.-T.S.); paul.sirbu@umfiasi.ro (P.-D.S.); 2 “St. Spiridon” County Clinical Emergency Hospital, 700111 Iasi, Romania; dancionca@yahoo.com (D.V.C.); dciupilan@gmail.com (D.C.); 3 Doctoral School of Biology, Faculty of Biology, “Alexandru Ioan Cuza” University of Iași, 700505 Iasi, Romania; malina.visternicu@yahoo.ro; 4 Department of Biology, Faculty of Biology, “Alexandru Ioan Cuza” University of Iași, 700505 Iasi, Romania; alin.ciobica@uaic.ro; 5 “Olga Necrasov” Center, Department of Biomedical Research, Romanian Academy, 700505 Iasi, Romania; 6 CENEMED Platform for Interdisciplinary Research, “Grigore T. Popa” University of Medicine and Pharmacy, 700115 Iasi, Romania

**Keywords:** biomechanics, clinical outcomes, ACL reconstruction, graft failure, graft selection, return to sport

## Abstract

Anterior cruciate ligament (ACL) reconstruction represents the current gold standard for restoring knee stability following ligament rupture, particularly in young and physically active individuals. The aim of this narrative review is to provide an updated overview of the biomechanical, biological, and clinical considerations underlying graft selection in ACL reconstruction, with particular emphasis on graft incorporation, fixation strategies, and postoperative outcomes. While surgical techniques and fixation devices have undergone substantial refinement, the optimal choice of graft remains a subject of ongoing debate. Traditionally, emphasis has been placed on the biomechanical characteristics of grafts, including tensile strength, stiffness, and resistance to cyclic loading. However, increasing attention has been directed toward biological factors that influence graft incorporation and clinical outcomes. The aim of this narrative review is to summarize the currently available literature regarding graft selection in ACL reconstruction by integrating biomechanical properties, biological graft incorporation, fixation strategies, and clinical outcomes. Particular emphasis is placed on the relationship between biomechanical performance and biological healing, as well as postoperative outcomes, in order to facilitate individualized graft selection in contemporary orthopedic practice.

## 1. Introduction

The anterior cruciate ligament (ACL) plays a central role in maintaining knee stability by controlling anterior tibial translation and rotational movements [1]. ACL rupture is one of the most common sports-related knee injuries, particularly among athletes participating in pivoting and cutting sports, and remains a significant clinical challenge due to functional impairment and the risk of long-term joint degeneration [1,2]. Despite advances in prevention strategies, ACL rupture continues to represent a significant clinical challenge due to functional impairment and risk of long-term joint degeneration [3].

The substantial number of ACL reconstructions performed worldwide further highlights the clinical burden of these injuries [4]. Reconstruction of the ACL has evolved considerably over the past decades, transitioning from non-anatomic techniques toward more refined procedures that aim to restore native ligament anatomy and biomechanics. Nevertheless, graft selection remains a critical factor influencing surgical success [5]. While early research focused primarily on mechanical strength and fixation methods, it has become increasingly evident that long-term outcomes depend on the biological processes that govern graft incorporation and remodeling [6].

Despite the substantial body of literature comparing graft options for ACL reconstruction, the optimal graft choice remains a matter of ongoing debate. Current controversies extend beyond biomechanical properties to include biological incorporation, donor-site morbidity, return-to-sport outcomes, revision risk, and patient-reported outcomes [7,8,9]. Moreover, increasing emphasis has been placed on individualized graft selection according to patient characteristics, activity level, and functional demands. Although several reviews have addressed individual graft types or specific clinical outcomes, a comprehensive synthesis integrating biomechanical evidence, biological remodeling, fixation strategies, and patient-specific considerations remains limited [8,9]. Addressing these aspects may facilitate more informed surgical decision-making and improve personalized treatment strategies.

The aim of this narrative review is to summarize the currently available literature regarding graft selection in ACL reconstruction by integrating biomechanical properties, biological graft incorporation, fixation strategies, and clinical outcomes. Particular emphasis is placed on the relationship between biomechanical performance and biological healing in order to facilitate individualized graft selection in contemporary orthopedic practice. Unlike previous reviews that primarily focus on individual graft characteristics or isolated clinical outcomes, the present review integrates biomechanical evidence, graft biology, fixation techniques, and patient-specific clinical considerations into a single comprehensive overview. Furthermore, recently introduced graft options are discussed alongside established grafts, providing an updated perspective on current trends in ACL reconstruction.

## 2. Methodology

This article was designed as a narrative review intended to summarize the current biomechanical and clinical concepts related to graft selection in anterior cruciate ligament reconstruction. A structured literature search was conducted in PubMed, Scopus, and Web of Science. Eligible studies were limited to those published between January 2000 and March 2026. The search combined terms including “ACL reconstruction”, “graft selection”, “bone–patellar tendon–bone”, “hamstring tendon”, “quadriceps tendon”, “peroneus longus tendon”, “allograft”, “synthetic graft”, “biomechanics”, “graft healing”, and “clinical outcomes”. Original clinical studies, biomechanical investigations, systematic reviews, meta-analyses, and relevant basic science studies published in English were considered. Studies unrelated to graft selection, animal studies without translational relevance, conference abstracts, editorials, and non-English publications were excluded. Titles and abstracts identified through the database searches were screened for relevance, and the full texts of potentially eligible articles were subsequently obtained and evaluated according to the predefined inclusion and exclusion criteria. The reference lists of relevant articles were also manually screened to identify additional studies not captured by the primary database search. A total of 118 references were ultimately selected based on their methodological quality, clinical relevance, and contribution to the narrative objectives of the review.

## 3. Graft Options in ACL Reconstruction

The selection of an appropriate graft for anterior cruciate ligament reconstruction represents one of the most critical determinants of both short-term mechanical stability and long-term biological success. Each graft type presents distinct biomechanical properties, healing characteristics, and donor-site implications [10,11]. Consequently, the decision must be individualized, taking into account patient-specific factors such as age, activity level, anatomical considerations, and risk tolerance. Beyond initial fixation strength, the biological process of graft incorporation, including revascularization and remodeling, plays a fundamental role in the ultimate functional outcome [12,13].

### 3.1. Bone–Patellar Tendon–Bone (BTB)

The bone–patellar tendon–bone graft is widely regarded as one of the most reliable options for ACL reconstruction, particularly in high-demand athletes. This graft consists of the central third of the patellar tendon along with bone blocks harvested from the patella and tibial tuberosity, allowing for direct bone-to-bone healing within the femoral and tibial tunnels [14]. This mode of integration is biologically advantageous, as it facilitates rapid osteointegration and provides a strong and stable fixation early in the postoperative period [15].

From a biomechanical perspective, the BTB graft demonstrates high stiffness and load-to-failure values that are comparable to, or slightly exceed, those of the native ACL. This contributes to excellent control of anterior tibial translation and rotational stability. Furthermore, the rigid fixation achieved through interference screws enhances initial stability, reducing micromotion at the graft–bone interface and promoting more predictable healing [10,16].

Despite these advantages, the use of BTB grafts is associated with specific drawbacks. Donor-site morbidity remains a significant concern, with patients frequently reporting anterior knee pain, particularly during activities involving kneeling or prolonged flexion [17]. Additionally, complications such as patellar fracture, patellar tendon rupture, and extensor mechanism dysfunction, although relatively rare, must be considered. These limitations have led to a more selective use of BTB grafts, particularly in patients for whom anterior knee symptoms may be problematic [18,19].

From a clinical perspective, BTB autografts continue to be regarded as the reference standard for young, highly active patients and competitive athletes because of their excellent long-term stability, lower graft failure rates, and high rates of return to sport [20].

Anterior knee pain, pain during kneeling, and extensor mechanism complications remain the principal disadvantages of BTB grafts. Although advances in graft harvesting techniques and postoperative rehabilitation have reduced these complications, they continue to occur more frequently than with hamstring or quadriceps tendon autografts [10]. Consequently, BTB grafts are generally preferred for competitive athletes involved in pivoting sports, whereas alternative grafts may be more appropriate for patients whose occupations or daily activities require frequent kneeling [10,21].

Current evidence suggests that graft selection should not rely solely on biomechanical superiority but rather on patient-specific characteristics, including age, activity level, occupational demands, previous knee surgery, and expectations regarding return to sport [22]. Although BTB grafts have traditionally been associated with superior objective knee stability, comparative studies have shown that functional outcomes and patient-reported outcome measures are often similar to those achieved with hamstring tendon grafts. These findings suggest that graft selection should be individualized according to patient characteristics rather than based solely on biomechanical considerations [23].

### 3.2. Hamstring Tendon Grafts

Hamstring tendon grafts, typically harvested from the semitendinosus and gracilis tendons, represent one of the most commonly used alternatives to BTB grafts. These tendons are usually folded into a multi-strand configuration, most often quadrupled, to achieve sufficient diameter and mechanical strength. The resulting construct exhibits high ultimate tensile strength, often exceeding that of the native ACL, and demonstrates favorable viscoelastic properties [24,25,26].

One of the primary advantages of hamstring grafts is the reduced donor-site morbidity compared to BTB grafts. Patients generally experience less anterior knee pain and a quicker return to activities that involve kneeling. Additionally, the surgical technique is less invasive, with smaller incisions and reduced disruption of the extensor mechanism [27,28].

However, the biological integration of hamstring grafts differs significantly from that of BTB grafts. Hamstring grafts undergo tendon-to-bone healing, which is generally slower than the bone-to-bone healing observed with BTB grafts and may influence the timing of graft incorporation and mechanical maturation [29]. In clinical practice, this difference should be considered when evaluating graft fixation, rehabilitation progression, and the potential for early graft motion within the bone tunnels. Furthermore, variability in graft diameter and fixation methods may influence mechanical performance and clinical outcomes [6]. Concerns have also been raised regarding potential graft elongation over time, which may contribute to residual laxity [30]. Despite these limitations, hamstring grafts remain a highly effective option, particularly in patients where minimizing donor-site morbidity is a priority.

### 3.3. Rectus Femoris Tendon

The rectus femoris tendon has recently been explored as an alternative autograft source for ACL reconstruction, representing a distinct harvesting option within the quadriceps musculotendinous complex [31]. The graft generally provides sufficient length and diameter for single-bundle reconstruction techniques, with satisfactory stiffness and resistance to cyclic loading. Preservation of the deeper quadriceps tendon layers has also been described as a feature of this harvesting technique [32]. The localized nature of rectus femoris tendon harvesting and preservation of surrounding tissue may contribute to reduced donor-site morbidity [33,34].

Preservation of continuity within the remaining quadriceps tendon may also support postoperative recovery of quadriceps function. These characteristics have been described as relevant considerations when evaluating the use of the rectus femoris tendon as an ACL graft [31].

Biological incorporation of the rectus femoris tendon graft follows the principles of tendon-to-bone healing, similar to other soft tissue grafts. This process involves fibrovascular integration and gradual remodeling within the bone tunnels, ultimately leading to the formation of a functional enthesis-like interface. However, because this technique remains relatively novel, long-term clinical data regarding graft survival, rotational stability, and comparative revision rates are still limited [31,35].

### 3.4. Quadriceps Tendon Graft

The quadriceps tendon graft, often referred to in the literature as originating from the rectus femoris component of the quadriceps mechanism, has gained increasing attention as a versatile and reliable option for ACL reconstruction [36]. This graft can be harvested with or without a bone block from the patella, providing flexibility in surgical technique and fixation strategy [37].

Biomechanically, the quadriceps tendon offers a large cross-sectional area and substantial collagen content, resulting in high load-to-failure values and favorable stiffness characteristics. These properties make it particularly suitable for patients requiring a robust graft, such as those undergoing revision surgery or those with larger anatomical dimensions [38,39].

From a clinical perspective, quadriceps tendon grafts are associated with lower rates of anterior knee pain compared to BTB grafts, while still providing strong fixation when a bone block is included. The donor-site morbidity is generally moderate and well tolerated, with preservation of extensor mechanism function in most cases [40,41].

The healing characteristics of quadriceps grafts depend on whether a bone block is used. When harvested as a soft tissue graft, integration follows a tendon-to-bone pathway similar to hamstring grafts. When a bone block is included, partial bone-to-bone healing may occur, potentially enhancing fixation strength and accelerating incorporation [42]. Although historically underutilized, increasing clinical evidence supports the quadriceps tendon as a valuable alternative that combines favorable biomechanical properties with acceptable morbidity.

Similar tendon-to-bone healing mechanisms are believed to occur after quadriceps tendon graft implantation, with progressive fibrocartilage formation and collagen reorganization contributing to long-term graft integration [4].

### 3.5. Peroneus Longus Tendon

The peroneus longus tendon has emerged as a promising alternative autograft for ACL reconstruction because of its favorable biomechanical properties, adequate graft dimensions, and relatively low donor-site morbidity, while also serving as a useful option in revision settings or when other graft sources are unsuitable [43,44].

The tensile strength and elasticity of the peroneus longus tendon are considered comparable to those of hamstring grafts, making it a viable substitute in terms of mechanical performance. Additionally, its length and diameter are generally sufficient to create a multi-strand construct suitable for ACL reconstruction [45,46].

A key concern associated with harvesting the peroneus longus tendon is the potential effect on ankle biomechanics, particularly in eversion strength and gait stability. However, clinical studies have suggested that compensatory mechanisms, including the function of the peroneus brevis muscle, may mitigate these effects, resulting in minimal long-term functional deficits [47,48].

### 3.6. Allografts

Allografts, derived from cadaveric donors, provide an attractive option in ACL reconstruction due to the absence of donor-site morbidity and reduced operative time. They are particularly useful in revision surgeries, multi-ligament injuries, or in patients who prefer to avoid autograft harvesting [49,50].

Despite these advantages, allografts present several biological and mechanical challenges. The processing methods required for sterilization, such as irradiation or chemical treatment, may compromise the structural integrity of the tissue, reducing its mechanical strength. Additionally, the absence of viable cells within the graft delays the processes of revascularization and cellular repopulation, resulting in slower incorporation and remodeling [51,52].

Reported clinical studies have described higher failure rates associated with allografts, particularly in younger and more active populations. This is likely due to a combination of reduced initial mechanical properties and delayed biological integration, which increases susceptibility to graft stretching or rupture during the early postoperative period [53,54]. Furthermore, although the risk is extremely low with modern screening techniques, the potential for disease transmission remains a theoretical concern. As a result, the use of allografts is generally reserved for specific indications where their advantages outweigh these limitations [55,56].

### 3.7. Synthetic Grafts

Synthetic grafts, such as those utilized in the Ligament Advanced Reinforcement System, were developed to overcome the limitations associated with biological grafts, particularly the need for graft harvesting and the prolonged period required for biological integration. These grafts are composed of industrial strength fibers designed to replicate the mechanical function of the ACL [57,58]. One of the primary advantages of synthetic grafts is the ability to provide immediate mechanical stability, allowing for early mobilization and accelerated rehabilitation. This is particularly beneficial in patients who require a rapid return to activity or in cases where biological graft options are limited [59,60].

However, the long-term performance of synthetic grafts remains a subject of concern. Complications such as synovitis, mechanical wear, and the generation of particulate debris have been reported, potentially leading to inflammatory reactions within the joint. Additionally, unlike biological grafts, synthetic materials do not undergo ligamentization, which limits their ability to adapt to physiological loading conditions over time [61]. Although newer generations of synthetic grafts have demonstrated improved outcomes compared to earlier designs, their use remains relatively limited and is generally reserved for carefully selected cases. Ongoing research aims to enhance their biocompatibility and integration with host tissues, potentially expanding their role in future ACL reconstruction strategies [57,62].

## 4. Biomechanical Comparison of Graft Types

The biomechanical comparison of graft types used in anterior cruciate ligament reconstruction represents a central aspect in understanding their clinical performance. Although numerous graft options are available, their evaluation requires a nuanced analysis that goes beyond simple measurements of ultimate tensile strength [63]. Parameters such as stiffness, viscoelastic behavior, fixation stability, resistance to cyclic loading, and biological remodeling must all be considered in order to accurately assess their functional equivalence to the native ligament [64,65].

At the time of implantation, most commonly used grafts demonstrate mechanical properties that equal or exceed those of the native ACL. However, this initial advantage is transient, as grafts undergo a process of biological remodeling characterized by cellular repopulation, revascularization, and structural reorganization [66]. During this phase, mechanical strength temporarily decreases before gradually recovering, a phenomenon that highlights the importance of both initial biomechanical properties and subsequent biological adaptation [67].

Stiffness is a critical parameter that influences joint kinematics and load distribution. Bone–patellar tendon–bone grafts consistently exhibit higher stiffness compared to soft tissue grafts, closely approximating that of the native ACL [68]. This contributes to superior control of anterior tibial translation, particularly in the early postoperative period. In contrast, hamstring tendon grafts generally display lower stiffness, which may result in increased anterior laxity, especially under cyclic loading conditions [10].

Quadriceps tendon grafts demonstrate biomechanical characteristics that position them between BTB and hamstring grafts. Their larger cross-sectional area contributes to high load-to-failure values, while their structural composition provides favorable resistance to elongation. These properties make them particularly suitable for patients requiring a robust graft without the increased donor-site morbidity associated with BTB harvest [69].

The behavior of grafts under cyclic loading is of particular importance, as it simulates the repetitive stresses experienced during daily activities and rehabilitation. Cyclic loading can lead to progressive elongation, commonly referred to as creep, which may compromise joint stability over time. Hamstring grafts have been shown to be more susceptible to elongation under repeated loading compared to BTB grafts, potentially due to differences in fixation methods and the viscoelastic properties of tendon tissue [70]. Conversely, bone-to-bone fixation in BTB grafts provides greater resistance to micro-motion, thereby reducing the risk of early graft slippage [71].

Fixation strength is another critical determinant of biomechanical performance. Interference screw fixation in BTB grafts allows for direct compression of bone plugs within the tunnels, resulting in high initial stability. In contrast, soft tissue grafts rely on fixation devices such as suspensory systems or interference screws, which may permit a small degree of micro-motion at the interface. This micro-motion can influence both mechanical stability and biological healing, particularly during the early postoperative period when the graft is most vulnerable [72,73].

Allografts present a distinct biomechanical profile, as their properties are influenced by processing techniques such as irradiation and chemical sterilization. These processes can reduce collagen integrity and alter viscoelastic behavior, leading to decreased strength and increased susceptibility to failure. Additionally, the delayed biological incorporation of allografts further compromises their mechanical performance over time, particularly in young and active individuals [74,75,76].

Synthetic grafts, such as those used in the LARS technique, exhibit very high initial stiffness and resistance to deformation. Their mechanical behavior remains relatively stable over time, as they do not undergo biological remodeling [77,78]. However, this lack of adaptation may represent a disadvantage, as the graft cannot respond to physiological loading conditions in the same manner as biological tissues. Furthermore, the mismatch between the mechanical properties of synthetic materials and native bone may lead to stress shielding or abnormal load distribution within the joint [79].

An important consideration in the biomechanical comparison of grafts is the interaction between mechanical properties and biological processes. While BTB grafts provide superior initial fixation and stiffness, their long-term success also depends on the quality of bone integration. Similarly, although hamstring and quadriceps grafts may exhibit slightly inferior initial stiffness, their outcomes are strongly influenced by the formation of a stable tendon–bone interface and the progression of ligamentization [7,80].

Overall, available literature (Table 1) indicates that no single graft type appears universally superior in all biomechanical aspects. Instead, each graft presents a unique combination of strengths and limitations that must be considered within the context of individual patient characteristics. A comprehensive understanding of these biomechanical differences, combined with an appreciation of the biological environment, is essential for optimizing graft selection and improving the long-term success of ACL reconstruction [81,82,83].

**Table 1:** Comparative Biomechanical Characteristics of ACL Grafts.

Parameter	BTB	Hamstring	Quadriceps	Allograft	Synthetic	References
Initial stiffness	Similar to native ACL	Generally lower than BTB	Intermediate to high	Variable depending on processing	Higher than biological grafts	[84,85]
Load to failure	Comparable to native ACL	Often exceeds native ACL	Comparable to BTB	Reduced after sterilization	Very high	[86]
Resistance to cyclic loading	Good	Moderate	Good	Reduced	Excellent	[87]
Fixation stability	Bone-to-bone fixation	Soft-tissue fixation dependent	Soft-tissue or hybrid fixation	Variable	Immediate mechanical fixation	[88]
Creep (elongation)	Low	Moderate	Low to moderate	Higher	Minimal	[89]
Biological adaptation	Rapid bone integration	Tendon-to-bone healing	Tendon-to-bone or mixed healing	Delayed incorporation	Absent	[90]

Abbreviations: ACL, anterior cruciate ligament; BTB, bone–patellar tendon–bone.

## 5. Graft Fixation Techniques and Initial Stability

Fixation strength is a critical determinant of biomechanical performance and represents one of the key factors influencing early graft stability, risk of micromotion, and biological incorporation after anterior cruciate ligament reconstruction [91]. The primary goal of fixation is to secure the graft within the bone tunnels while minimizing displacement under cyclic loading during early rehabilitation, a period in which the graft is mechanically vulnerable before biological integration is complete [6].

In BTB grafts, fixation is achieved primarily through interference screw systems that allow direct compression of bone plugs within the femoral and tibial tunnels [92]. This bone-to-bone contact provides a high level of primary stability, reduced micromotion at the graft–bone interface, and favorable conditions for early osteointegration. The rigid fixation obtained with interference screws also allows early controlled mobilization protocols due to the predictable mechanical behavior of the construct [16].

In contrast, soft tissue grafts such as hamstring and quadriceps tendons rely on suspensory fixation devices or interference screws without bone blocks. Suspensory fixation provides strong initial fixation strength but introduces a longer graft-to-bone healing distance, which may delay biological integration [93,94]. Additionally, these constructs are more susceptible to micro-motion at the tendon–bone interface, particularly under cyclic loading conditions during early rehabilitation [95].

Micro-motion, although sometimes limited, has important biological implications. Excessive relative movement at the graft–bone interface may interfere with the formation of a stable fibrocartilaginous transition zone, which is essential for tendon-to-bone healing [96]. This can potentially prolong the ligamentization process and increase the risk of tunnel widening or graft elongation in the early postoperative phase [97].

Another important aspect of fixation biomechanics is load-to-failure behavior. BTB constructs generally demonstrate higher initial stiffness and lower elongation under cyclic loading compared to soft tissue grafts. However, advances in fixation technology, including adjustable-loop cortical fixation devices and hybrid fixation techniques, have significantly improved the mechanical performance of soft tissue grafts, narrowing the gap between graft types in terms of initial stability [98,99,100].

## 6. Clinical Outcomes

### 6.1. Return to Sport

Return to sport after ACL reconstruction is a multifactorial process influenced by graft type, biological healing, neuromuscular recovery, psychological readiness, and rehabilitation protocols [101,102].

Several studies have reported that premature return to sport significantly increases the risk of re-injury, particularly in young athletes [103]. For this reason, many authors advocate delayed return-to-sport timelines, often extending up to 9–12 months or even longer in high-risk populations. In some cases, return may be delayed up to two years depending on graft maturation and functional performance criteria [12].

Objective criteria used to guide return to sport decisions include limb symmetry index, strength testing, hop performance, and neuromuscular control assessments [104].

### 6.2. Graft Failure and Revision Rates

Graft failure remains one of the most important complications following ACL reconstruction and is influenced by patient age, activity level, graft type, and fixation method. Younger, highly active individuals are at significantly higher risk of re-rupture due to increased exposure to high-demand sports and incomplete biological maturation of the graft [105].

Allografts have consistently demonstrated higher failure rates compared to autografts in young and active populations [54]. This increased risk is attributed to delayed biological incorporation, reduced initial cellular viability, and potential weakening of graft structure due to processing methods such as irradiation [54,106].

Autografts such as BTB, hamstring, and quadriceps tendons generally show lower revision rates, although each graft type presents specific risk profiles. BTB grafts are associated with lower failure rates in high-demand athletes, while hamstring grafts may demonstrate slightly higher rates of residual laxity and elongation in some studies [107].

Revision ACL reconstruction is technically more complex and is associated with worse functional outcomes compared to primary reconstruction, emphasizing the importance of optimal graft selection during the initial surgery [108].

### 6.3. Complications and Donor-Site Morbidity

Complications following ACL reconstruction can be broadly divided into graft-related complications, fixation-related issues, and donor-site morbidity. Among these, donor-site morbidity remains a major concern when autografts are used [109].

BTB grafts are particularly associated with anterior knee pain, especially during activities involving kneeling, squatting, or prolonged knee flexion. This pain is thought to result from disruption of the extensor mechanism and patellar tendon harvesting site. Additionally, complications such as patellar fracture, patellar tendon rupture, and extensor mechanism weakness, although rare, have been reported [17,19].

Hamstring grafts generally demonstrate lower rates of anterior knee pain but may be associated with hamstring weakness, particularly in deep flexion or high-speed sprinting activities. Quadriceps tendon grafts show intermediate donor-site morbidity, with some patients reporting anterior thigh discomfort but overall good functional tolerance [110].

Allografts eliminate donor-site morbidity entirely but introduce concerns related to slower biological incorporation and higher failure risk in young patients. Synthetic grafts avoid donor-site complications but are associated with potential inflammatory reactions and long-term mechanical degradation [111].

## 7. Patient-Specific Factors in Graft Selection

Graft selection in anterior cruciate ligament reconstruction is not universal and must be individualized according to patient-specific factors that influence both mechanical demands and biological healing capacity [10].

Age is one of the most important determinants, as younger patients exhibit higher activity levels and greater risk of graft failure. In this population, autografts with strong biomechanical properties, such as BTB or quadriceps tendon grafts, are often preferred due to their lower revision rates [112].

Activity level and type of sport are also critical. High-demand pivoting sports such as football, basketball, and skiing require grafts with high initial stiffness and resistance to cyclic loading. In contrast, recreational athletes may tolerate grafts with slightly different biomechanical profiles [22].

Anatomical factors, including tunnel size, tendon diameter, and knee morphology, also influence graft choice. For example, patients with smaller hamstring tendons may not achieve adequate graft diameter, increasing failure risk [113].

Previous surgeries represent another key factor, particularly in revision ACL reconstruction where primary graft options may no longer be available [114].

Finally, patient expectations and willingness to accept donor-site morbidity play an important role in decision-making. Some patients may prioritize avoidance of anterior knee pain, favoring hamstring or quadriceps grafts over BTB [115].

## 8. Current Gaps in the Literature

Despite considerable advances in ACL reconstruction, several important knowledge gaps remain. Long-term comparative studies evaluating newer graft options, particularly the rectus femoris and peroneus longus tendons, are still limited. Likewise, the biological mechanisms governing graft incorporation and maturation have not been completely elucidated, and considerable heterogeneity exists regarding rehabilitation protocols and outcome measures across clinical studies. Future well-designed prospective multicenter trials combining clinical, biomechanical, and biological endpoints are required to establish evidence-based recommendations for individualized graft selection.

## 9. Discussions

ACL reconstruction has evolved substantially over the past decades, and multiple graft options have demonstrated favorable clinical outcomes. However, no single graft has proven universally superior, making graft selection a patient-specific decision that should consider biomechanical performance, biological incorporation, donor-site morbidity, activity level, and individual expectations [24,71]. The native ACL provides an important biomechanical reference for this decision, as its viscoelastic behavior and high tensile strength contribute to its ability to withstand anterior tibial translation and rotational loading. Therefore, the mechanical properties and biological behavior of the selected graft should be considered in relation to the functional demands of the native ligament [91,116,117,118].

BTB autografts remain the preferred option for many young, highly active patients because of their excellent stability and lower graft failure rates, although they are associated with increased anterior knee pain and donor-site morbidity. Hamstring tendon autografts offer lower donor-site morbidity but may present slightly higher residual laxity in some patient groups, while quadriceps tendon autografts have emerged as a reliable alternative with promising clinical outcomes. Peroneus longus tendon grafts and allografts may be appropriate in selected clinical situations, although long-term evidence remains limited and allografts have shown higher failure rates in young, active individuals. Synthetic grafts continue to evolve, but concerns regarding long-term durability and biological integration persist [14,67,93].

Current evidence increasingly supports individualized graft selection rather than a universal approach. Patient age, activity level, sporting demands, associated injuries, and expectations should guide graft choice. Emerging concepts, including biological augmentation and patient-reported outcome measures, may further improve decision-making, although additional high-quality studies with long-term follow-up are still required to establish definitive recommendations [4,9,103].

## 10. Future Research Directions

Despite the large number of comparative studies, important controversies remain regarding the optimal graft for different patient populations, long-term graft survival, biological remodeling, and the role of emerging biological augmentation strategies. Furthermore, heterogeneity among published studies limits direct comparisons and highlights the need for standardized outcome measures.

Future research should focus on the development of personalized graft selection algorithms, long-term prospective comparative studies, standardized patient-reported outcome measures, and the clinical effectiveness of biological enhancement techniques. In addition, further investigation of novel graft options, biomaterials, and strategies to improve graft incorporation may contribute to more individualized treatment approaches and improved long-term outcomes following ACL reconstruction.

## 11. Conclusions

ACL reconstruction remains the gold standard for restoring knee stability following ACL rupture; however, graft selection continues to represent one of the most important determinants of postoperative outcomes. Although BTB, hamstring tendon, quadriceps tendon, allografts, peroneus longus tendon, and synthetic grafts each present specific biomechanical and biological advantages, none has demonstrated universal superiority across all clinical scenarios.

Current evidence supports an individualized approach to graft selection based on patient age, activity level, sporting demands, occupation, associated injuries, previous surgical history, and expectations regarding return to sport. High-demand athletes may benefit from the excellent stability and graft durability associated with BTB autografts, whereas hamstring and quadriceps tendon grafts offer effective alternatives with lower donor-site morbidity. Allografts and synthetic grafts should be reserved for carefully selected clinical indications because of their specific limitations.

## Data Availability

No new data were created or analyzed in this study. Data sharing is not applicable to this article.
